# Changes in Physical Meat Traits, Protein Solubility, and the Microstructure of Different Beef Muscles during Post-Mortem Aging

**DOI:** 10.3390/foods9060806

**Published:** 2020-06-19

**Authors:** Yong-Hong Feng, Song-Shan Zhang, Bao-Zhong Sun, Peng Xie, Kai-Xin Wen, Chen-Chen Xu

**Affiliations:** Institute of Animal Sciences, Chinese Academy of Agricultural Sciences, Beijing 100093, China; yike556@163.com (Y.-H.F.); zhangsongshan@caas.cn (S.-S.Z.); sunbaozhong@caas.cn (B.-Z.S.); seulbird@163.com (P.X.); wenkaixin215@126.com (K.-X.W.)

**Keywords:** beef muscle, protein solubility, myofibril fragmentation, microstructure, aging

## Abstract

This study was performed to compare the differences in pH, myofibril fragmentation index (MFI), total protein solubility (TPS), sarcoplasmic protein solubility (SPS), myofibrillar protein solubility (MPS), and the microstructure of seven beef muscles during aging. From the six beef carcasses of Xinjiang brown cattle, a total of 252 samples from *semitendinosus* (ST), *longissimus thoracis* (LT), *rhomboideus* (RH), *gastrocnemius* (GN), *infraspinatus* (IN), *psoas major* (PM), and *biceps femoris* (BF) muscles were collected, portioned, and assigned to six aging periods (1, 3, 7, 9, 11, and 14 day/s) and 42 samples were used per storage period. IN muscle showed the highest pH (*p* < 0.05) from 1 to 14 days and the lowest TPS (*p* < 0.01) from 9 to 14 days with respect to the other muscles. Moreover, the changes in IN were further supported by transmission electron microscopy due to the destruction of the myofibril structure. The highest value of MFI was tested in ST muscle from 7 to 14 days. The total protein solubility in PM, RH, and GN muscles were not affected (*p* > 0.05) as the aging period increased. The lowest TPS was found in the RH muscle on day 1, 3, and 7 and in the IN muscle on day 9, 11, and 14. The pH showed negative correlations with the MFI, TPS, and MPS (*p* < 0.01). The results suggest that changes in protein solubility and muscle fiber structure are related to muscle location in the carcass during aging. These results provide new insights to optimize the processing and storage of different beef muscles and enhance our understanding of the biological characteristics of Xinjiang brown cattle muscles.

## 1. Introduction

Meat and meat products are important nutrient-intensive foods that are widely consumed worldwide [1]. For consumers, tenderness is a critical indicator that drives their willingness to repurchase and acceptability [2,3]. There are still great variations in the tenderness between different parts of beef muscles [4,5]. In the beef industry, aging is often considered to be one of the important factors determining the ultimate tenderness of the meat [6,7]. Due to the differences between the biochemical characteristics of these muscles, they may cause different degrees of response to aging [4]. Therefore, the inconsistency of the tenderness of different muscles has become the main problem that currently exists in the meat processing industry during the aging process.

Reducing the intake of fat in meat is essential for maintaining consumer health and reducing the risk of illness [8]. In fact, the type of muscle fiber is one of the factors that affects the lean meat rate [9]. The muscle fiber type differs greatly among various body parts and is generally divided into slow-oxidized fiber (type I), fast oxido-glycolytic fiber (type IIA), and fast glycolytic fibers (type IIB and type IIX) [10,11]. Studies indicated that muscle fiber type was closely associated with muscle quality and more type IIB fiber might lead to poorer muscle quality and lower lean meat productivity [12,13]. Our previous unpublished study has shown that s*emitendinosus* (ST) and *longissimus thoracis* (LT) muscles of Xinjiang brown cattle have large percentages of type IIB fibers (approximately 47.89% and 40.85%, respectively), *rhomboideus* (RH) and *gastrocnemius* (GN) muscles have high percentages of type IIA fibers (approximately 61.43% and 42.10%, respectively), and *infraspinatus* (IN), *psoas major* (PM), and *biceps femoris* (BF) muscles have large percentages of type I fibers (79.69%, 43.06%, and 39.75%, respectively). In the current study, we focused on these seven muscles, representing the characteristics of the three muscle fiber types.

Protein in skeletal muscle is a key factor in determining the type of muscle fiber. Establishing the relationship between muscle protein structure and functional properties will contribute to the understanding of the modification mechanism of meat products during processing and storage [14]. According to the solubility and location of protein, it can be divided into myofibrillar protein, sarcoplasmic protein, and matrix protein, which maintain the structural integrity of myofibrillar fiber [15,16]. These proteins are susceptible to oxidative degradation during aging, which may explain the meat tenderization after muscle fiber breakage [17]. Oxidation affects the chemical properties of proteins and changes in protein solubility can reflect the degree of protein denaturation [18,19]. Sarcoplasmic protein solubility (SPS) is sometimes used as a measure of muscle quality [20]. It has been well documented that precipitated or denatured sarcoplasmic proteins may bind to myofibrils, leading to a decrease in water holding capacity [21,22,23]. It is vital to the production of Xinjiang brown beef, for the lives of Xinjiang people, and for local meat production. However, there are still limited studies examining the changes in protein solubility between various parts of muscles of Xinjiang brown cattle during post-mortem aging.

Therefore, the aim of this research was to evaluate the changes in pH, myofibril fragmentation index (MFI), total protein solubility (TPS), SPS and myofibrillar protein solubility (MPS), and the microstructure of seven beef muscles (ST, LT, RH, GN, IN, PM, BF) during aging from 1 to 14 days.

## 2. Materials and Methods

### 2.1. Animals and Muscle Samples Preparation

Six Xinjiang brown bull calves of approximately 30 months of age were slaughtered at a local slaughterhouse (Yining, Xinjiang, China) according to the commercial procedures. The mean weight at slaughter was 566 ± 32 kg. All corn-fed calves were raised on the same farm to ensure background consistency and then slaughtered using electrical stunning on the same day. The carcasses were overhung in a cold room (3 ± 1 °C) for 24 h. All procedures were undertaken following the guidelines given by the Animal Care and Ethics Committee for animal experiments, Institute of Animal Science, Chinese Academy of Agricultural Sciences.

The *semitendinosus* (ST), l*ongissimus thoracis* (LT), *rhomboideus* (RH), *gastrocnemius* (GN), *infraspinatus* (IN), *psoas major* (PM), and *biceps femoris* (BF) muscles were collected from each carcass. The samples were kept on ice and transported to the laboratory after 50 min and all visible intermuscular and subcutaneous fat was removed. Prior to packaging, each sample was stamped with a date mark. Each muscle was further separated into three equal-length sections, resulting in six muscle sections (5 cm × 3 cm × 2 cm, 50 ± 0.05 g) per carcass, with 42 slices per aging period (six replications per muscle and per aging period). Afterwards, the samples were individually vacuum-packaged in a polyolefin bag and randomly assigned to aging at 3 ± 1 °C (relative humidity: 70–80%) for 1, 3, 7, 9, 11, and 14 days (within muscles, samples from the same carcass were not aged for the same time). Upon completion of each postmortem aging period, samples were taken to determine pH and transmission electron microscopy (TEM). The remaining samples were stored at −30 °C for further analysis within 2 weeks.

### 2.2. pH Measurement

The pH value of the beef muscles was measured at 1, 3, 7, 9, 11, and 14 days postmortem following the method described by Kim et al. [24], with minor modifications. Chopped meat (10 g) was mixed with 100 mL of distilled water for 15 s and then homogenized using an Ultra-Turrax T25 homogenizer (IKA-Werke, Gmbh & Co., Staufen in Breisgau, Germany) at 2800 g. The pH of the homogenate was measured using a pH meter equipped with an electrode (PB-10, the precision was 0.01, made by Sartorius Group, Goettingen, Germany). Each sample was determined three times.

### 2.3. Myofibril Fragmentation Index (MFI)

MFI was determined by modification of the method by Hou et al. [25]. Two gram samples were homogenized with a homogenizer (FJ200-S, Shanghai Specimen Model Co., Shanghai, China) at 10,000 g for 60 s (3 × 20 s with a 60 s break between bursts) at 4 ± 2 °C in 20 mL ice-cold buffer (100 mM KCl, 20 mM K_2_HPO_4_, 1 mM EGTA, 1 mM MgCl_2_, and 1 mM NaN_3_, pH 7.0). The homogenates were centrifuged using a LG10-24A model centrifuge (Peking Medical centrifuge Factory, Beijing, China) at 3000 g for 15 min at 4 °C and the supernatant was discarded. The pellets were homogenized in 20 mL of homogenizing buffer and centrifuged and the supernatant was discarded again. The resulting pellets were then resuspended in 5 mL of homogenizing buffer and filtered through a polyethylene strainer (200-mesh) to remove the fat and connective tissue. Then, 5 mL buffer was used to promote the passage of myofibrils through the strainer. The protein concentration of suspension was determined by the biuret method [26]. The protein concentration was diluted to 0.5 mg/mL and measured spectrophotometrically at 540 nm (UV 6100, Metash, Shanghai, China). MFI was calculated by multiplying A540 by 200.

### 2.4. Protein Solubility

The total protein solubility (TPS) and sarcoplasmic protein solubility (SPS) were measured using the procedure of Li et al. [27]. A 1 g sample of each muscle (six replications per muscle and per aging period) was homogenized with 10 mL cold 0.025 M potassium phosphate buffer (pH 7.2), which was then shaken at 4 °C for 12 h and centrifuged at 1500 g for 20 min. The total protein concentrations of the supernatants were assessed using the biuret method [26]. The step of SPS detection was that the muscle sample (1 g) was homogenized in ice-cold 20 mL 0.025 M potassium phosphate buffer (pH 7.2) and then carried out using the same shaking, homogenization, and centrifugation procedures mentioned above. Myofibrillar protein solubility (MPS) was calculated by TPS minus SPS. The value was expressed as micrograms of soluble protein per gram of meat.

### 2.5. Microstructure Analysis

At each storage time point, strips (approximately 5 × 1 × 1 mm) were cut off from three muscles (ST, RH, and IN) and were selected using transmission electron microscopy (TEM). Muscle samples were fixed with 2.5% glutaraldehyde in 0.1 M phosphate buffer (pH 7.2) and 2% osmium tetroxide. Then, the gradually increasing concentrations of ethanol (50–100%, *v/v*) were used for dehydration. The solvent was then switched to acetone and the samples were dehydrated with acetone gradient, followed by replacing with propylene oxide and being embedded with resin. The samples were trimmed, sectioned (50–70 nm), and stained with uranyl acetate and lead citrate. The samples were viewed under TEM (Hitachi, H-7500, Japan) at required magnification as per the standard procedures.

### 2.6. Statistical Analysis

The experimental design was a randomized complete block design, where each carcass (*n* = 6) served as a block. Each measurement was performed in triplicate. To determine the significant effect by two factors (muscles and aging period), data analysis was performed using the SAS 9.2 program (SAS Institute, Cary, NC, USA) with muscles and aging period as the main effects, using two-way analysis of variance (ANOVA). Analysis of variance was performed on all the variables using the General Linear Model (GLM) procedure. Duncan’s multiple range test (*p* < 0.05) was used to determine the significance of the differences in the mean values for different samples. Pearson’s correlation coefficients were calculated for variables.

## 3. Results and Discussions

### 3.1. Changes in pH Value

The changes in pH values of beef muscles during aging are shown in Table 1. There was a considerable variation in pH between muscles. Compared to the IN muscle, the pH values in ST, LT, PM, and BF were lower at 1 day (*p* < 0.05). The difference in pH may be due to the rate of glycolysis between muscles [28]. Similar results were obtained in some studies of beef and lamb [29,30], indicating that the rate of decrease in the pH of PM accelerated. Aging had no significant impact on pH in PM muscles (*p* > 0.05) from 1 to 14 days. Except for IN and PM, no significant changes were observed (*p* > 0.05) in the pH of the remaining six muscles from 3 to 14 days of aging. In terms of value, the pH of IN showed the highest value (*p* < 0.05) of all the muscles in the whole aging period. IN had more type I muscle fibers and its main characteristics were higher oxidative metabolism and lower glycogen content [31]. Therefore, in this study, we can speculate that lower glycolysis capacity would result in a higher pH of IN muscle, which might influence proteolysis.

### 3.2. Changes in Myofibril Fragmentation Index (MFI) 

Changes in MFI of different beef muscles during aging are displayed in Table 2. The highest MFI was observed in the RH muscle at 1 day (*p* < 0.05). The ST muscle had the highest MFI from day 7 onwards (*p* < 0.05). Compared with the other muscles, the MFI values of IN and LT were found to be lower from 3 to 11 days (*p* < 0.05). MFI is used as an important indicator of I band rupture and interstitial fibril connection breakage [32]. These results seem to indicate that the degree of rupture of muscle fibers in the I band is greater in ST muscles during aging, resulting in changes in the integrity and solubility of myofibrillar proteins [33,34]. The MFI of each individual muscle increased significantly with the aging period (*p* < 0.05) and this is in agreement with Li et al. [35]. However, the difference in MFI change was very small in the later days of the aging period. This might be due to the fact that as the aging period increases, the degree of variation in myofibril breaks becomes smaller [36].

### 3.3. Changes in Protein Solubility

The results of the changes in TPS, SPS, and MPS during aging are shown in Table 3. No significant changes (*p* > 0.05) were evidenced in TPS of RH, GN, and PM muscles and in MPS of IN and BF muscles during aging. In regard to SPS, there were no significant differences in SPS between different muscles at day 11 and 14 (*p* > 0.05). IN muscle exhibited a lower TPS at day 9, 11, and 14 than that at day 7 (*p* < 0.05). LT muscles had the highest TPS and MPS among all muscles at day 14 (*p* < 0.05). The SPS of RH was significantly lower than that of IN and GN at day 7 and 9 (*p* < 0.05). Protein solubility was an important indicator of protein properties, which was attributed to protein denaturation [37]. In this study, the results indicated that IN muscle had a high extent of protein denaturation after 9 days [19]. The solubility of myofibrillar protein increased with the aging period up to day 7 and then did not change significantly. This is related to the characteristics of myofibrillar proteins [38,39]. During aging, myofibrillar protein bonds were weakened and more protein hydrolysis was released, which resulted in higher MPS [40]. However, myofibrillar proteins are gradually unfolded as the aging period increases, exposing hydrophobic groups and resulting in almost no change in MPS [41].

### 3.4. Pearson Correlations

Correlation coefficients among pH, MFI, TPS, SPS, and MPS of all muscles during aging are presented in Table 4. MFI, TPS, and MPS are negatively correlated with pH (*p* < 0.01). MFI is positively correlated with MPS (*p* < 0.05), whereas no correlations with TPS and SPS are observed (*p* > 0.05). The correlation coefficients of TPS with MPS are higher than those of SPS, indicating that TPS was affected to a larger extent by the denaturation of MPS than that of SPS. These results suggest that pH is a more important determinant for protein solubility than MFI in this study. Moreover, the correlation can further explain the results of protein solubility and confirm that the variation in protein solubility of different muscles may be due to pH changes.

### 3.5. Changes in Muscle Microstructure

**Figure 1:**
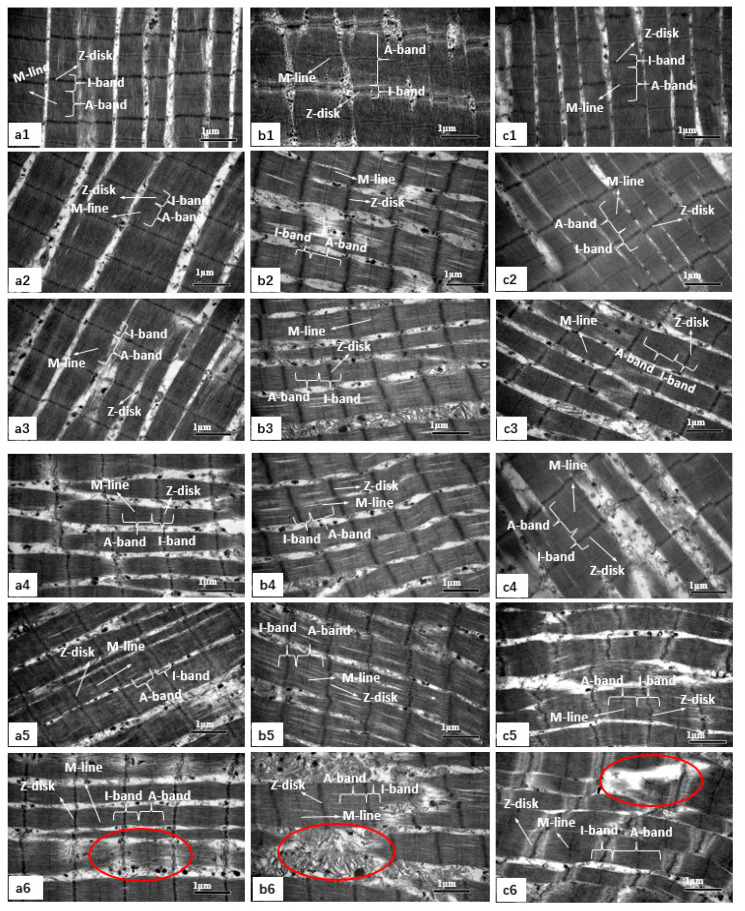
Transmission electron microscopy (TEM) images of beef muscles during different aging periods. TEM images at original magnifications of ×25,000. (**a1**–**a6**): The microstructure of *semitendinosus* (ST) at day 1, 3, 7, 9, 11, and 14. (**b1**–**b6**): The microstructure of *rhomboideus* (RH) at day 1, 3, 7, 9, 11, and 14. (**c1**–**c6**): The microstructure of *infraspinatus* (IN) at day 1, 3, 7, 9, 11, and 14. Red circles indicate the fracturing zones.

The changes in the microstructure of muscle (ST, RH, and IN) by TEM are shown in Figure 1. In ST, RH, and IN muscles, the sarcoplasmic reticulum around the sarcomeres can be clearly distinguished and the myofibrils are tightly combined with the visible I-band and A-band and the Z-disk and M-line can be differentiated on day 1 and day 3 (Figure 1(a1,a2,b1,b2,c1,c2)). After day 7, the overall integrity of the myofibrils diminishes (Figure 1(a3,b3,c3)). The M-line located on the centre of the A-band looks vague and the Z-disk and I-band junctions are weakened after day 9. The Z-disk is distorted but undamaged, although some longitudinal splits are evident in RH muscles (Figure 1(b4,b5)). The worst myofibrillar structure is observed in IN muscle, in which the overlapping structure of thick and thin filaments is destroyed, the skeletal muscle structure is severely broken, and the Z-disk is distorted and weakened (Figure 1(c4,c5)). At day 14, IN muscle shows fractures in the Z-disk, as well as fragmentation at the junction of the I-band and Z-disk (Figure 1(c6)). Pan and Yeh [42] found that cracking of muscle fibers and shortening of sarcomere could largely reduce the tenderness of the meat. Aside from mechanical damage, muscle fiber structure was also affected by endogenous proteases during aging [43,44]. Moczkowska et al. [45] had reported that BF muscles were more sensitive to oxidation than LL muscles and the extent of this phenomenon depends on the type of muscle examined. In this study, TEM results showed that IN muscle had a longer sarcomere length and a greater degree of rupture in the muscle fiber structure, as expected because this muscle has a higher proportion of type I fiber (approximately 79.69%), which predicted that the aging rate would be faster. These results are in accordance with the MFI described above. On the other hand, previous research shows that the concentration of coenzyme Q10, carnosine, and taurine in IN muscle was higher than that in LT muscle [46], implying a higher concentration of functional bioactive compounds in oxidized muscle. These results suggest that oxidized muscle accelerates the improvement of meat tenderness.

## 4. Conclusions

In this study, it can be concluded that the meat characteristics of different beef muscles responded differently to the storage time and were significantly affected by carcass position. The meat quality parameters of IN muscle were improved, which was due to its higher pH value, lower MFI and TPS, and greater degree of myofibril rupture, followed by ST and RH. However, there is little difference in meat quality parameters among other muscles. Therefore, for IN, it can be suggested that the characteristics are particularly suitable for producing high-quality beef products. Additionally, a significant interaction between pH and protein solubility was observed. Further research should be conducted to explore the mechanism of how pH affects protein solubility (for example, to assess its effect on cathepsin activity).

## Figures and Tables

**Table 1:** Changes in pH values of different beef muscles during aging.

Items	Aging (Days)						SEM
	1	3	7	9	11	14	
ST	5.65 ^a,y^	5.45 ^ab,x^	5.54 ^b,xy^	5.50 ^a,xy^	5.45 ^ab,x^	5.38 ^a,x^	0.028
LT	5.64 ^a,y^	5.38 ^a,x^	5.41 ^a,x^	5.39 ^a,x^	5.37 ^a,x^	5.40 ^ab,x^	0.027
RH	5.76 ^ab,y^	5.55 ^bc,x^	5.53 ^b,x^	5.62 ^ab,x^	5.56 ^b,x^	5.55 ^ab,x^	0.028
GN	5.72 ^ab,y^	5.46 ^ab,x^	5.53 ^b,x^	5.51 ^a,x^	5.38 ^a,x^	5.45 ^ab,x^	0.032
IN	5.87 ^b,z^	5.66 ^c,xy^	5.70 ^c,xy^	5.76 ^b,yz^	5.73 ^c,xyz^	5.56 ^b,x^	0.029
PM	5.59 ^a^	5.51 ^ab^	5.53 ^b^	5.58 ^ab^	5.51 ^ab^	5.55 ^ab^	0.022
BF	5.67 ^a,y^	5.45 ^ab,x^	5.42 ^a,x^	5.47 ^a,x^	5.43 ^ab,x^	5.51 ^ab,x^	0.026
SEM	0.026	0.023	0.022	0.038	0.030	0.022	

ST, *semitendinosus*; LT, *longissimus thoracis*; RH, *rhomboideus*; GN, *gastrocnemius*; IN, *infraspinatus*; PM, *psoas major*; BF, *biceps femoris*. ^a–c^ Values within a column with different superscript are significantly different (*p* < 0.05). ^x–z^ Values within a row with different superscript are significantly different (*p* < 0.05). SEM, standard error of the mean. *n* = 6 in each muscle at each aging period.

**Table 2:** Changes in the myofibril fragmentation index (MFI) of different beef muscles during aging.

Items	Aging (days)						SEM
	1	3	7	9	11	14	
ST	102.57 ^d,x^	107.93 ^c,x^	154.13 ^d,y^	157.53 ^e,y^	156.50 ^e,y^	189.93 ^e,z^	7.47
LT	81.87 ^bc,x^	83.10 ^a,x^	86.97 ^a,xy^	88.27 ^a,xy^	88.90 ^a,xy^	92.60 ^a,y^	1.17
RH	122.23 ^e,x^	129.93 ^d,xy^	131.73 ^c,xy^	132.97 ^cd,xy^	133.77 ^c,xy^	143.43 ^cd,y^	2.11
GN	87.97 ^c,x^	105.87 ^c,y^	128.56 ^c,z^	131.70 ^c,z^	131.37 ^c,z^	136.93 ^c,z^	4.41
IN	68.80 ^a,x^	76.87 ^a,xy^	79.77 ^a,xy^	84.37 ^a,y^	87.63 ^a,y^	101.83 ^ab,z^	2.62
PM	75.90 ^ab,x^	93.47 ^b,y^	98.90 ^b,y^	111.87 ^b,z^	110.93 ^b,z^	110.07 ^b,z^	3.27
BF	99.43 ^d,w^	122.87 ^d,x^	130.67 ^c,x^	141.80 ^d,y^	144.33 ^d,yz^	152.47 ^d,z^	4.33
SEM	3.95	4.19	5.77	5.76	5.62	7.07	

ST, *semitendinosus*; LT, *longissimus thoracis*; RH, *rhomboideus*; GN, *gastrocnemius*; IN, *infraspinatus*; PM, *psoas major*; BF, *biceps femoris*. ^a–c^ Values within a column with different superscript are significantly different (*p* < 0.05). ^x–z^ Values within a row with different superscript are significantly different (*p* < 0.05). SEM, standard error of the mean. *n* = 6 in each muscle at each aging period.

**Table 3:** Changes in protein solubility of different beef muscles during aging.

Items	Aging (days)						SEM
	1	3	7	9	11	14	
**Total Protein Solubility, mg/g**							
ST	172.77 ^ab,x^	190.83 ^bc,yz^	207.59 ^c,z^	183.39 ^ab,xy^	192.81 ^ab,yz^	193.16 ^bc,yz^	3.19
LT	181.08 ^bcd,x^	198.36 ^c,xy^	193.45 ^b,xy^	195.42 ^ab,xy^	200.21 ^b,y^	200.06 ^c,y^	2.42
RH	169.00 ^a^	174.24 ^a^	176.05 ^a^	183.80 ^ab^	181.94 ^ab^	185.91 ^ab^	2.25
GN	189.47 ^d^	187.76 ^abc^	196.72 ^bc^	198.46 ^b^	185.01 ^ab^	194.29 ^bc^	2.01
IN	176.24 ^abc,x^	185.24 ^abc,x^	195.85 ^bc,y^	178.47 ^a,x^	177.82 ^a,x^	179.02 ^a,x^	1.95
PM	183.20 ^cd^	191.27 ^bc^	187.48 ^ab^	184.91 ^ab^	187.58 ^ab^	189.43 ^abc^	1.30
BF	179.64 ^bc,x^	179.67 ^ab,x^	208.62 ^c,y^	183.42 ^ab,x^	186.39 ^ab,x^	187.92 ^ab,x^	2.94
SEM	1.69	2.25	2.67	2.31	2.48	1.77	
**Sarcoplasmic Protein Solubility, mg/g**							
ST	59.71 ^abc,xy^	57.20 ^ab,xy^	60.66 ^b,y^	49.57 ^ab,x^	50.90 ^xy^	50.53 ^xy^	1.51
LT	68.31 ^c,y^	65.49 ^b,y^	50.83 ^a,x^	48.75 ^ab,x^	51.99 ^x^	51.51 ^x^	2.17
RH	51.35 ^a,xy^	59.33 ^ab,y^	48.77 ^a,x^	42.00 ^a,x^	46.14 ^x^	44.02 ^x^	1.70
GN	62.45 ^bc,yz^	58.45 ^ab,yz^	67.01 ^b,z^	55.05 ^b,xy^	48.67 ^x^	55.15 ^xy^	1.73
IN	55.69 ^ab,yz^	52.04 ^a,xy^	62.61 ^b,z^	52.65 ^b,xy^	45.06 ^x^	46.51 ^xy^	1.76
PM	51.93 ^a,y^	59.08 ^ab,z^	47.68 ^a,x^	48.06 ^ab,x^	47.51 ^x^	44.08 ^w^	1.21
BF	55.29 ^ab,x^	60.24 ^ab,xy^	70.66 ^b,y^	48.20 ^ab,x^	49.23 ^x^	57.48 ^xy^	2.31
SEM	1.55	1.19	2.14	1.15	1.02	1.68	
**Myofibrillar Protein Solubility, mg/g**							
ST	113.07 ^a,x^	133.63 ^b,y^	146.93 ^b,y^	133.81 ^ab,y^	141.91 ^y^	142.63 ^abc,y^	3.28
LT	112.77 ^a,x^	132.87 ^b,y^	142.62 ^ab,y^	146.67 ^b,y^	148.22 ^y^	148.55 ^c,y^	3.59
RH	117.66 ^ab,x^	114.90 ^a,x^	127.28 ^a,xy^	141.80 ^ab,y^	135.80 ^y^	141.89 ^abc,y^	3.10
GN	127.01 ^de,x^	129.31 ^b,xy^	129.71 ^a,xy^	143.41 ^ab,y^	136.34 ^xy^	139.14 ^abc,xy^	2.15
IN	120.55 ^bc^	133.20 ^b^	133.23 ^ab^	125.82 ^a^	132.75	132.52 ^ab^	1.91
PM	131.27 ^e,x^	132.18 ^b,x^	139.80 ^ab,xy^	136.85 ^ab,xy^	140.06 ^xy^	145.34 ^bc,y^	1.62
BF	124.35 ^cd^	119.44 ^ab^	137.96 ^ab^	135.22 ^ab^	137.16	130.44 ^a^	2.55
SEM	1.57	2.09	2.17	2.37	2.33	1.86	

ST, *semitendinosus*; LT, *longissimus thoracis*; RH, *rhomboideus*; GN, *gastrocnemius*; IN, *infraspinatus*; PM, *psoas major*; BF, *biceps femoris*. ^a–c^ Values within a column with different superscript are significantly different (*p* < 0.05). ^x–z^ Values within a row with different superscript are significantly different (*p* < 0.05). SEM, standard error of the mean. *n* = 6 in each muscle at each aging period.

**Table 4:** Pearson correlation coefficients for pH, MFI, and protein solubility.

	MFI	TPS	SPS	MPS
pH	−0.314 **	−0.419 **	−0.032	−0.377 **
MFI		0.086	−0.144	0.183 *
TPS			0.300 **	0.743 **
SPS				−0.416 **

MFI, myofibril fragmentation index; TPS, total protein solubility; SPS, sarcoplasmic protein solubility; MPS, myofibrillar protein solubility. * *p* < 0.05; ** p < 0.01. *n* = 6 in each muscle at each aging period.
